# *Imagine the Possibilities Pain Coalition* and Opioid Marketing to Veterans: Lessons for Military and Veterans Healthcare

**DOI:** 10.3390/healthcare13040434

**Published:** 2025-02-18

**Authors:** Christopher K. Haddock, Luther Elliott, Andrew Kolodny, Christopher M. Kaipust, Walker S. C. Poston, Jennifer D. Oliva, Eleanor T. Lewis, Elizabeth M. Oliva, Nattinee Jitnarin, Chunki Fong

**Affiliations:** 1 NDRI-USA, Inc., 1920 West 143rd Street, Suite 120, Leawood, KS 66224, USA; kaipust@ndri-usa.org (C.M.K.); poston@ndri-usa.org (W.S.C.P.); jitnarin@ndri-usa.org (N.J.); fong@ndri-usa.org (C.F.); 2 School of Global Public Health, New York University, 708 Broadway, 6th Floor, New York, NY 1003, USA; luther@nyu.edu; 3 Heller School for Social Policy and Management, Brandeis University, 415 South Street, Waltham, MA 02453, USA; akolodny@brandeis.edu; 4 Maurer School of Law, Indiana University, 211 S. Indiana Ave., Bloomington, IN 47405, USA; jenoliva@iu.edu; 5 Program Evaluation and Resource Center, Center for Innovation and Implementation, Department of Veterans Affairs, 795 Willow Road, Menlo Park, CA 94025, USA; eleanor.lewis@va.gov (E.T.L.); elizabeth.oliva@va.gov (E.M.O.)

**Keywords:** veterans, military, chronic pain, opioid crisis, pharmaceutical industry, marketing practices

## Abstract

**Background/Objectives**: The opioid crisis has disproportionately impacted U.S. military veterans, who face heightened risks of opioid use disorder and overdose due to chronic pain and mental health conditions. The pharmaceutical industry’s role in misrepresenting opioid risks—leading to over USD 50 billion in legal settlements—has included targeted marketing to vulnerable populations. This study examines Janssen Pharmaceuticals’ “Imagine the Possibilities Pain Coalition” (IPPC), which aimed to increase opioid use among veterans with chronic non-cancer pain. Insights from this public health industry document analysis offer guidance for military medicine and healthcare policymaking. **Methods**: Using the Opioid Industry Document Archive (OIDA), housed at Johns Hopkins University and the University of California, San Francisco, researchers conducted retrospective content analysis. Documents referencing veterans were identified through keyword searches on Johns Hopkins’ SciServer portal and reviewed using CoCounsel, an AI-based legal document platform *using a human-in-the-loop* approach. Relevant documents were examined by the authors to extract material aligned with the research focus. **Results**: The IPPC employed strategies to influence opioid prescribing for veterans. These included educational materials that minimized addiction risks and exaggerated long-term benefits and empathy-driven narratives prioritizing immediate pain relief over potential harms. Ghostwriting ensured favorable perspectives on opioids in scientific literature, aligning with broader industry strategies to promote opioids for chronic pain. **Conclusions**: The targeted marketing of opioids to veterans has exacerbated the opioid crisis, as documented in government reports and litigation. Rigorous oversight of industry-funded coalitions and evidence-based practices are critical to insulating military healthcare from corporate influence and addressing the opioid crisis among veterans.

## 1. Introduction

The opioid epidemic is a major public health crisis, marked by a sharp rise in prescription and illicit opioid use, leading to substantial morbidity and mortality. Opioid-related overdose deaths rose from 8050 in 1999 to 84,411 in 2022 [1,2,3]. The crisis’s roots trace back to the late 1990s and early 2000s, when aggressive marketing of opioid analgesics drove a dramatic increase in prescribing, subsequently fueling an increase in the prevalence of opioid use disorder and overdose [4]. Although heroin and synthetic opioids like fentanyl now dominate overdose cases, the surge in prescription opioids laid the foundation for the epidemic [5]. Research shows that even medically supervised prescription use for chronic, non-cancer pain can result in opioid use disorder (OUD) and a transition to illicit opioids [6]. Veterans have been particularly impacted, facing overdose mortality rates nearly double those of the general population during the peak period of opioid prescribing in the United States (U.S.) [7,8,9,10].

In response to evidence of deceptive marketing practices, the pharmaceutical industry has faced over USD 50 billion in legal settlements, among the largest in U.S. history [11,12]. Johnson & Johnson (J&J; through Janssen Pharmaceuticals), the focus of this research, agreed to pay USD 5 billion in a nationwide settlement [13], following a USD 572 million judgment in Oklahoma [14]. In the Oklahoma case, District Court Judge Thad Balkman ruled that J&J aggressively marketed opioids and minimized addiction risks, contributing to the crisis in the state. Reports from the United States *President’s Commission* [15], the *National Academies* [16], and the *Stanford–Lancet Commission* [17] similarly concluded that aggressive marketing and misrepresentation of opioids for chronic non-cancer pain led to overprescribing and subsequent addiction and overdose.

Documents of litigation involving tobacco companies, JUUL, Vioxx, pharmaceutical manufacturers, and several other industries are available through the University of California, San Francisco [18], or Johns Hopkins University [19] websites. These documents provide insights into marketing strategies and corporate communications that resulted in negative public health outcomes. Published research based on documents of litigation analyzes internal corporate documents, such as correspondence, marketing strategies, and policy memos, and is made public through litigation or investigations. These documents serve as the primary data and are analyzed qualitatively by investigators to uncover themes, strategies, patterns of behavior, and their implications for healthcare and public health. Over 1000 publications leveraging litigation documents have informed health policies and exposed marketing practices that led to negative public health outcomes [18,20]. This precedent underscores the need for research based on the Opioid Industry Document Archive (OIDA), which has been widely encouraged by academic groups [21,22]. Several case studies and narrative reviews based on opioid litigation documents have already been published in the literature [23,24,25,26].

Yakubi, Gac, and Apollonio [26,27] reported evidence from OIDA documents demonstrating that J&J targeted vulnerable populations to increase opioid sales, including young children, the elderly, and veterans. Building on their work, this paper provides a comprehensive review and case study focused on Janssen Pharmaceutical’s “Imagine the Possibilities Pain Coalition” (IPPC) and its promotion of opioid use among veterans. The IPPC is a lesser-known industry initiative compared to others, such as the now-defunct American Pain Foundation’s Military/Veteran Initiative and the production of the popular book *Exit Wounds* [28]. By analyzing internal documents related to the IPPC’s targeting of veterans, this study aims to extract critical lessons for providers serving veterans and the broader healthcare system worldwide.

## 2. Materials and Methods

This study was funded by a grant from the U.S. National Institute of Drug Abuse (NIDA R01DA058016) and involves analysis of documents of discovery from the publicly available OIDA, housed at both Johns Hopkins University [19] and the University of California, San Francisco [29]. For this study, we extracted all OIDA documents that referenced veterans or the military through the Johns Hopkins SciServer portal [30]. The Johns Hopkins SciServer allows users to analyze OIDA’s metadata, documents, and extracted text using Jupyter Python notebooks in a web-based environment, eliminating the need for additional software or large data downloads. Required notebooks are provided for tasks such as querying and downloading documents and performing basic text analysis and visualization. Users with basic Python knowledge can customize these notebooks. Notebooks our team customized for this research can be obtained from the first author (C.K.H.). We conducted comprehensive keyword searches related to veterans or the military for all documents contained in the archive as of March 2024. In total, we extracted 30,988 documents referencing veterans and 30,182 referencing the military from the archive. These documents were bulk downloaded onto the NDRI-USA cloud server for use in this study. Detailed instructions on accessing documents through SciServer are available from Johns Hopkins on their “OIDA Resources” site [19]. Python notebooks customized for this study can be attained for the first author for large downloads (C.K.H.).

These documents were uploaded into the Artificial Intelligence (AI) platform *CoCounsel* (Thomson Reuters, Toronto, ON, Canada) to assist in identifying and summarizing relevant discovery documents, which included both structured materials (e.g., marketing presentations) and unstructured communication (e.g., emails) [31,32,33]. CoCounsel was built upon OpenAI’s GPT-4 with training and fine-tuning conducted using extensive legal datasets [34]. Tools such as CoCounsel represent a significant advance over traditional models of document identification (e.g., snowball sampling), where researchers manually review documents until concluding that a sufficient sample has been obtained. AI improves this methodology by rapidly analyzing large document archives, detecting implicit connections and patterns beyond predefined keywords, and ensuring comprehensive and scalable identification of relevant documents. The document archive was uploaded from the NDRI-USA cloud server into CoCounsel, creating a centralized library for analysis.

We utilized a “human-in-the-loop” approach, in which human reviewers independently read and analyzed each identified document identified by CoCounsel, using AI-generated summaries to ensure no relevant content was overlooked. CoCounsel prompts were used iteratively to search and summarize documents referencing the IPPC and military/veterans or related information. For example, a typical prompt would be “Identify and summarize documents referring to the ‘Imagine the Possibilities Pain Coalition.’ Provide an overview of their mission, key people involved, initiatives, projects, with a focus on targeting military veterans”. Similar prompts were used to explore related topics, including affiliated companies and IPPC authors’ papers. Extracted materials relevant to the IPPC mission and activities, including visual presentations (four of which appear as figures in this paper), served as the primary data for this paper.

Copies of litigation document data were initially reviewed and analyzed qualitatively by the primary authors (C.K.H., L.E.). Objective findings regarding the IPPC’s mission and activities, as well as key themes describing their approach to marketing opioids to veterans and broader implications for healthcare and public health, were developed using an interpretative approach [35,36]. Materials were also evaluated from other sources (e.g., media reports, magazine articles, published literature) to supplement the analyses. Key documents included in this paper and the qualitative findings of the primary authors were reviewed collaboratively by all authors, a group that included military veterans (C.K.H., W.S.C.P.), experts on opioid use disorder and overdose (A.K. and L.E.), opioid litigation (A.K., J.D.O.), pain management and opioid therapy in the US Veterans Administration (E.L.T., E.M.O.), and occupational epidemiology and data science (C.M.K., N.J., C.F.). Additionally, one author (W.S.C.P.) contributed lived experience as a veteran with chronic pain and related disability treated through the Veterans Administration. This collective expertise ensured a comprehensive evaluation of the IPPC’s marketing strategies while grounding the findings in practical, clinical, and public health contexts, further informed by lived experience as a veteran with chronic pain.

## 3. Results

### 3.1. Description and Vision of the Imagine the Possibilities Pain Coalition

Industry documents analyzed establish that, in 2011, Robyn Kohn, the National Advocacy Director for Johnson & Johnson Health Care Systems, Inc., sent an email to their health policy and advocacy leads that included a briefing document announcing a new initiative—the “Imagine the Possibilities Pain Coalition IPCC-Janssen Pharmaceuticals” [37]. The IPPC was to include members of the Janssen pain teams and external members from the “pain communities”. The goal of the coalition was to “align and address issues in pain management with emphasis on abuse and diversion”. The launch date of the IPPC was 24 July 2011.

**Figure 1:**
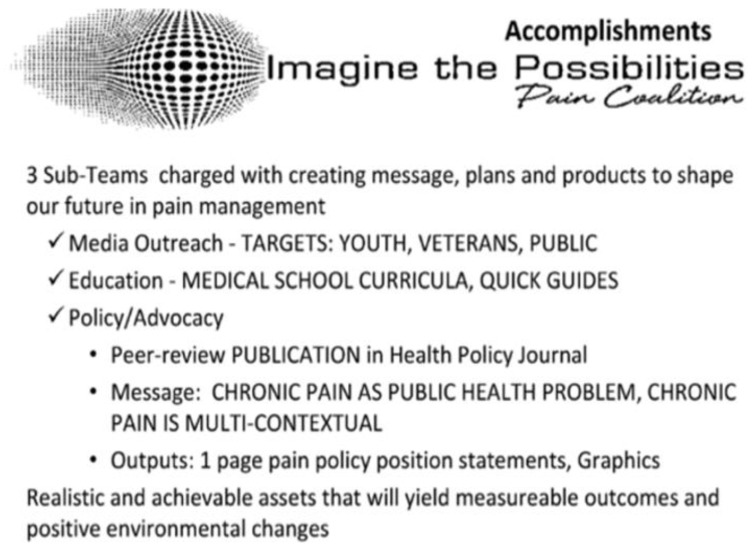
IPPC Vision.

A directory of the original members of the IPPC can be found in the OIDA document archive [38]. Of the 19 members, 9 were Janssen employees, while the remaining members were from industries aligned with the pharmaceutical sector, and four were from academia or an academic medical center. Figure 1 is an excerpt from an internal Janssen Pharmaceuticals document outlining the vision of the IPPC: “To create a broad-based community in the field of Pain Management focused on influencing the pain narrative to be person-with-pain centered, and, therefore, more holistically managed”. This marketing narrative promotes an approach that emphasizes the patient with chronic non-cancer pain’s current symptoms and preferences in treatment decisions and aligns with the coalition’s stated “priority area of focus” on the “epidemic of pain” rather than the “epidemic of addiction” [39].

**Figure 2:**
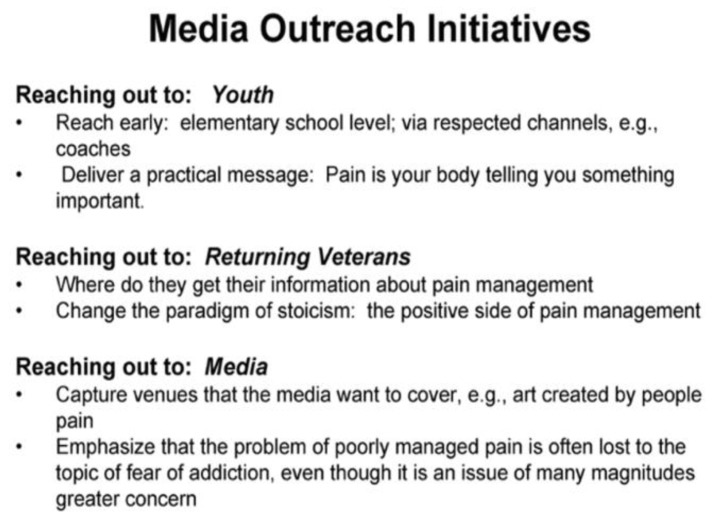
IPPC Media Outreach Initiatives.

Figure 2 lists the media outreach initiatives of the IPPC [39]. For veterans, the two primary messaging goals noted in Figure 2 were to influence where they sought information about pain management and to change the “paradigm of stoicism”. To achieve this, the IPPC outreach team was tasked with producing educational materials targeting both providers and the public. An underlying theme of these materials was that “the problem of poorly managed pain is often lost to the topic of fear of addiction, even though it is an issue of many magnitudes of importance”.

### 3.2. IPPC Leverages Veteran Pain Narratives to Promote Opioids for Chronic Pain

**Figure 3:**
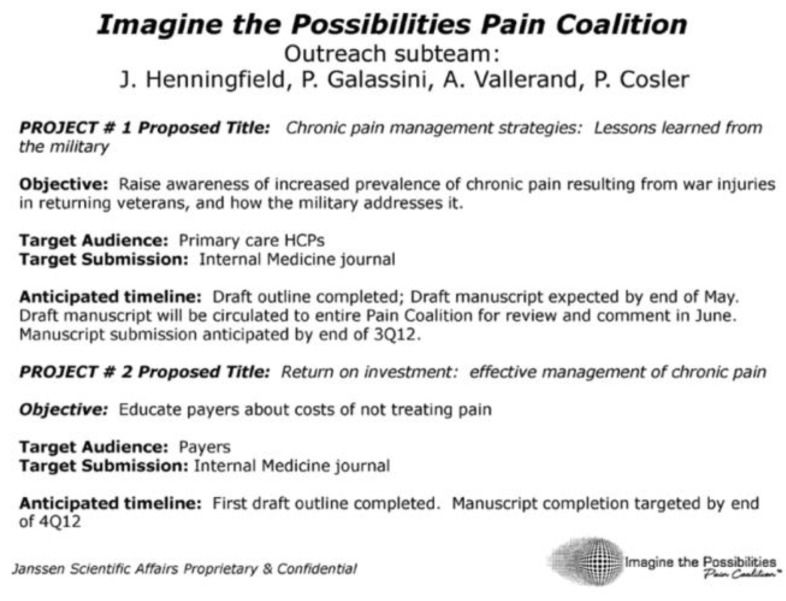
IPPC Manuscript Project.

In the Janssen Pharmaceutical industry document “Update & 2013 National Advocacy Business Planning” from August 2012, an IPPC outreach sub-team proposed a project to “raise awareness of increased prevalence of chronic pain resulting from war injuries in returning veterans, and how the military addressed it” [40]. This goal was to be accomplished by producing a scientific paper, authored by IPPC members including Janssen employees, titled “Chronic Pain Management Strategies and Lessons from the Military: A Narrative Review” (henceforth “Vallerand et al”, see Figure 3). The article appropriately addresses the issue of chronic pain among military personnel and veterans, highlighting efforts to improve available services. However, a central message of the paper was that long-term opioids have been successfully used for non-cancer, chronic pain patients in the military; thus, applying this strategy to the general U.S. population might lead to more effective pain management and improved long-term patient outcomes. This article has been cited by others, with references to it appearing as late as 2024 [41]. An early draft of the article can be found in the OIDA archives [42]. The paper was reviewed by Coalition members and was eventually published in the journal *Pain Research and Management* under the title “Pain Management Strategies and Lessons from the Military: A Narrative Review” [43] notably omitting the original emphasis on chronic pain in the title.

The Vallerand et al. paper [43] exemplifies two common strategies employed by the pharmaceutical industry to influence the prescription of opioid therapy for chronic pain patients in general and veterans specifically:

(1) Ghostwriting to ensure that published papers convey messages beneficial to the industry;

(2) Creating positive expectations for and downplaying the risks of long-term opioids for non-cancer chronic pain, which has also been noted by Yakubi et al. [26].

#### 3.2.1. Ghostwriting and Conflicts of Interest 

**Figure 4:**
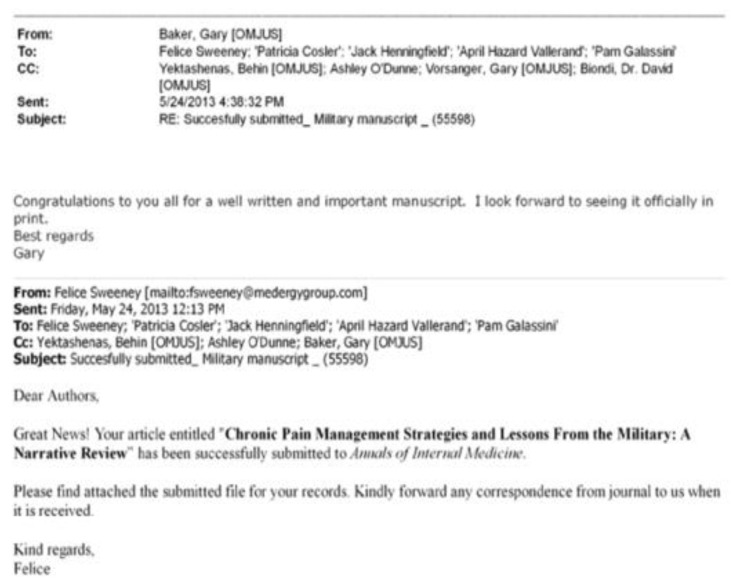
Email to Authors from MedEgry.

MedErgy, a medical marketing company frequently mentioned in the OIDA archives as being contracted to assist with the development of papers on opioid products, provided manuscript development support for the IPPC. The paper acknowledges MedErgy’s involvement in its writing: “Editorial support for manuscript preparation was provided by Megan Knagge, PhD, of MedErgy, and was funded by Janssen Scientific Affairs, LLC.”. An email from Felice Sweeny of MedErgy (Figure 4) to the authors indicates that final article processing and submission was handled by MedErgy [44]. 

The acknowledgment in the paper states, “The authors retained full editorial control over the content of the manuscript.” The interpretation of “full editorial control” is not clear from the document, given previous research demonstrates that, in other articles prepared by MedErgy, this acknowledgment was used even when the paper’s authors raised concerns about claims that were ultimately retained in the paper [45,46,47]. One of the authors of the Vallerand et al. paper, Patricia Cosler, was an employee of Janssen. Despite being employed by Janssen, a company with a vested interest in marketing opioids, the paper’s disclosure section claimed that Cosler “declares no current conflicts of interest”. Similarly, the other authors, all consultants to Janssen, declared no conflicts of interest.

#### 3.2.2. Creating Positive Expectations and Downplaying Risks 

Prominent themes of the Vallerand et al. paper include the following: that opioids are an effective treatment for chronic pain and that the serious adverse effects of long-term opioid use can be managed by stratifying patients based on claimed risk factors and by monitoring so-called high-risk patients more closely. For example, in the abstract of the paper, the authors write that “opioids are regarded as a critical part of acute and chronic pain management schemes” (p. 261). In table (Table 1 [43]) and the related discussion, the authors claim that patients can be stratified and managed based on pre-determined risk factors. The authors acknowledge the rising misuse of opioids among active-duty service members and veterans while countering that “measures have been taken to address this issue, including the development of a landmark structured program, the Opioid Renewal Clinic (ORC), to assist primary care providers at an urban veterans hospital who are managing patients with chronic non-cancer pain that requires opioid analgesia” (p. 265). Notably, data from the ORC at the Philadelphia VA Medical Center reported a 51% rate of aberrant drug-taking behaviors among their patients, with 32.2% of these cases remaining unresolved after intervention [48]. In a paper published contemporaneously with the Vallerand et al. article, [49] the same VA ORC staff noted that patients exhibiting aberrant behaviors were tapered off opioids, and the goal for others was to reduce opioid doses due to the significant risks of overdose and adverse drug effects. 

The Vallerand et al. paper claims that pain management programs in the military seek to “improve the long-term, monitored use of opioid analgesics” (p. 263), citing a paper by Cleeland and colleagues [50]. However, the cited paper specifically discusses pain management programs within the Veterans Administration and does not mention opioids or opioid products. This risks giving the impression that Cleeland et al. explicitly support the expanded use of opioids in military pain management. Similarly, Vallerand et al. state that “Opioid analgesics are recommended for patients who experience severe or disabling pain that is insufficiently controlled with acetaminophen or NSAIDs” and reference an article by Chou and colleagues [51]. The recommendation in the Chou paper stressed the risks of opioid use: “Because of substantial risks, including aberrant drug-related behaviors with long-term use inpatients vulnerable or potentially vulnerable to abuse or addiction, potential benefits and harms of opioid analgesics should be carefully weighed before starting therapy.”

To properly contextualize its recommendations, it is important to compare the position advocated in the Vallerand et al. paper with the understanding of the risks of long-term opioid therapy for chronic pain at the time. In 2010, well before the publication of the Vallerand paper, the *VA/DoD Guideline for Opioid Therapy for Chronic Pain* acknowledged the potential harms of opioids and their limited long-term benefits for chronic pain management, recommending non-pharmacological and non-opioid pharmacological modalities as first-line treatments [52]. By 2015, a substantial body of evidence had demonstrated the risks associated with long-term opioid use for non-cancer pain [53,54,55,56,57]. It was well known that opioid abuse prevalence was almost seven times higher in the veteran’s administration population than in commercial health plans, [58] with high-dose use associated with major depression and post-traumatic stress disorder [59]. Evidence existing in 2015 from the VA indicated that long-term opioid therapy for chronic pain carried an increased risk of serious harm, which was dose-dependent [60]. The development of tolerance was a significant issue with long-term use, and dose escalation could lead to opioid-induced hyperalgesia, exacerbating rather than relieving pain [61].

## 4. Discussion

The opioid crisis has profoundly impacted U.S. military veterans, a group disproportionately affected due to the high prevalence of chronic pain and mental health conditions. Unlike the tobacco industry, the pharmaceutical industry contributes to life-saving medical advancements; however, the opioid crisis highlights that even companies that manufacture products to improve health can engage in harmful practices in an effort to increase profits. Our analysis of industry documents reveals targeted marketing strategies aimed at veterans with chronic non-cancer pain, which included downplaying risks and overstating benefits. These efforts involved funding educational materials, ghostwriting research to minimize addiction concerns, and promoting opioids as vital for pain management. While the IPPC aimed to enhance pain treatment for veterans, it also sought to boost opioid use. Key lessons for military medicine and the broader medical community follow.

### 4.1. Scrutinize the Composition and Objectives of Public Health Coalitions

A U.S. Senate investigative committee concluded that pharmaceutical companies, aiming to increase opioid sales, funded outreach groups and public health organizations to create messaging that highlighted the benefits of their products while downplaying the risks [62]. Among the most well-known organizations that focused on opioids was the American Pain Society (APS), which was leveraged by Purdue Pharmaceuticals and other opioid manufacturers, due to their marketing efforts [4]. Prior to 2019, the APS was perceived as a reputable professional society in the US, with notable individuals in pain management actively involved in its efforts. However, it was forced to close in 2019 and has faced lawsuits due to its involvement with the pharmaceutical industry and its role in the opioid crisis [63].

A similar group, the American Pain Foundation (APF), described itself as the nation’s largest organization for pain patients. The APF disbanded in 2012 after investigations revealed that it received 90% of its funding in 2010 from the pharmaceutical and medical-device industries and that its treatment guides downplayed the risks associated with opioids while overstating their benefits [64]. The APF engaged in extensive outreach to influence veterans, including funding the book and website *Exit Wounds* [28], which promoted the benefits of opioids for chronic non-cancer pain and the Military/Veteran Pain Initiative [64,65]. The IPPC had a similar purpose to the APS and APF, with a goal of marketing opioids to children and veterans.

Considering the widespread influence and potential adverse impacts of industry-funded public health advocacy groups, healthcare systems and providers should carefully evaluate the composition of public health advocacy organizations and coalitions, particularly when these groups receive industry funding or include members with financial ties to life sciences companies.

### 4.2. Be Sensitive to the Possibility of Trojan Marketing

It is crucial to recognize when the stated goals of such coalitions, like promoting “person-with-pain centered” approaches, might be aligned with increasing the use of specific drugs or devices, including opioids. This would be an example of *trojan horse marketing*, which presents a product or strategy with a seemingly beneficial message to gain consumer trust, while subtly promoting additional aspects that primarily benefit the company (e.g., increased sales) [66,67]. While the IPPC vision of “more holistically managed” care appears commendable, the promotion of the “person-with-pain” narrative can plausibly be seen as a trojan horse marketing strategy that downplays evidence-based care by emphasizing the patient’s subjective experience, potentially leading to a preference for short-term symptom management over considerations of long-term risks.

One example of how a seemingly beneficial initiative may be aligned with marketing goals not in the best interest of patients is the APS’s “Pain as the 5th Vital Sign” initiative. Although ostensibly aimed at ensuring that patients’ pain was adequately recognized and treated, it resulted in the overuse of opioids [63]. Some physicians, fearing punitive measures for inadequate pain management, felt compelled to prescribe opioids, sometimes at the risk of patient health [68]. In 2001, the Joint Commission on Accreditation of Healthcare Organizations implemented standards requiring healthcare providers to record patients’ reported pain scores as a vital sign - an objective measure considered critical to life. By 2004, the “vital sign” terminology was removed from the accreditation standards manual in response to criticisms that it encouraged opioid use, yet the requirement for aggressive monitoring of a patient’s subjective pain score remained in place [69]. However, there are growing calls to stop treating pain as the fifth vital sign because of its hypothesized role in exacerbating the opioid crisis [70].

A marketing narrative that was foundational to the IPPC mission was to “challenge the paradigm of Stoicism”. Although a “paradigm of stoicism” might be understood as the stoic philosopher Epictetus’ dictum in *The Enchiridion* to ignore what is outside of our control (e.g., “grin and bear it”) without seeking care, strategies derived from stoic philosophy—such as focusing on controllable aspects of pain, challenging negative thoughts, regulating emotions, accepting pain without undue distress, and viewing challenges as opportunities for growth—form the basis of Cognitive Behavioral Therapy for Chronic Pain (CBT-CP), the primary evidence-based behavioral treatment used in the VA [71,72,73]. CBT-CP encourages patients to accept their pain as part of their lived experience, focus on what they can control (e.g., their thoughts, behaviors, and attitudes), and cultivate resilience [72,73]. One goal of CBT-CP is to reduce reliance on opioids among chronic pain patients. Taken together, downplaying concerns about addiction and disregarding psychological strategies to manage pain could serve as an impactful messaging strategy to increase the use of opioid therapy by providing an immediate solution that providers were led to believe was safe.

### 4.3. Promote Evidence-Based Practices over Marketing Narratives

Empathy-driven and person-centered clinical narratives, while ostensibly compassionate, can also be marketing strategies to boost drug sales, including opioids. This marketing message may lead physicians to de-emphasize or overlook the potential harm associated with the product, as the initial positive framing may distract attention from concern about serious adverse effects. Another marketing narrative in the Vallerand et al. paper is that chronic pain patients can be stratified and managed based on pre-determined risk factors for OUD. Similar to empathy-driven and person-centered narratives, the message that the risks of OUD can be effectively predicted and managed may lower the perceived risks of long-term opioid use among physicians, leading to increased sales. However, the medical evidence does not support the long-term use of opioids for chronic non-cancer pain, and it does not support the use of risk stratification tools and strategies [74]. 

### 4.4. Be Vigilant When Approving Educational Offerings

Industry-supported medical education initiatives are aligned with the sponsoring company’s marketing objectives [75]. For instance, Persaud uncovered that industry-sponsored materials promoting the effectiveness of long-term opioid use for non-cancer pain, while downplaying the associated risks, were integrated into Canadian medical school curricula [76]. These materials were backed by pharmaceutical companies marketing opioids and were presented by speakers with financial ties to those companies, without full disclosure of conflicts of interest. Healthcare systems should ensure that their educational programs present a balanced view of the risks and benefits associated with all treatment options, thereby mitigating the potential for biased or incomplete information.

### 4.5. Be a Critical Consumer of Scientific Literature

Several researchers and scientific organizations have identified that pharmaceutical companies leveraged biased and misrepresented peer-reviewed publications to downplay the risks and exaggerate the benefits of opioids, contributing significantly to the opioid crisis [77,78]. For example, the World Health Organization retracted two of its publications that promoted aggressive opioid use [79] and admitted that, after an internal review, the guidelines were unduly influenced by the pharmaceutical industry [80]. Medical practitioners and educators should remain vigilant about the potential biases in published research, particularly for literature that specifically aims to influence the practices of healthcare providers.

Ghostwriting is an important issue for healthcare practitioners to be aware of as consumers of medical literature. Ghostwriting in medical literature refers to the practice where professional writers draft scientific papers that are then attributed to researchers who may have had little to no involvement in the writing or research [81,82,83]. According to a 2009 internal industry document titled “Tapentadol Team Status” [47], MedErgy, the company that worked with the IPPC on the Vallerand et al. paper, typically billed about USD 30,200.00 for each manuscript (approximately USD 48,433.00 in 2024 dollars), with additional costs totaling around USD 2025.00 per paper. Gac and colleagues [84] noted that this document tracked the development of 12 manuscripts for publication in academic journals. One project, titled “State of the Art: Multimodal Therapy for Chronic Pain”, showed that the original author had withdrawn, with the next step noted as securing approval of an outline from a new author. Of the twelve projects, seven involved the company either designing or drafting the study before sending it to the listed authors for review [47]. Thus, the phrase “editorial support” may underrepresent the contribution of ghostwriters for manuscripts. Research has shown that this system of paid ghostwriters has resulted in a substantial number of papers in the scientific literature echoing industry messages [84].

Healthcare professionals should also be aware of important conflicts of interest (COIs) in medical literature. COIs arise when professional judgment concerning a primary interest, such as the effectiveness and risks of opioids, is influenced by a secondary interest, such as financial gain [85]. According to Fugh-Berman [86], in many industry-funded papers, once ghostwriters write articles embedding the sponsor’s marketing message, the completed articles are then approved by paid guest authors, usually academically affiliated physicians, who may amend them but typically avoid edits that conflict with the sponsor’s goals. The guest author submits the manuscript as their original work, and any requested revisions from the journal are handled by the medical writer for the author’s signature. This process creates obvious COIs—an author is paid to serve as the author to increase its perceived credibility. The presence of COIs can lead to biased research outcomes and a loss of public trust in medical findings. 

Despite clear COIs, the authors of the Vallerand et al. article declared that they had no COIs. One of the authors, Henningfield, declared no COIs in another article titled “Negative outcomes of unbalanced opioid policy supported by clinicians, politicians, and the media” [87]. This article, like Vallerand’s, promotes the benefits of long-term opioid use for chronic non-cancer pain. The declaration of interest in the original paper stated that “The authors report no conflicts of interest”. After a reader familiar with the author’s work for opioid manufacturers complained that the Declaration of Interest was flawed and the authors had significant conflicts of interest, the following was published by the journal: “Erratum: In issue 30(1) of the *Journal of Pain & Palliative Care Pharmacotherapy*, an error appeared in the Declaration of Interest for the article ‘Negative Outcomes of Unbalanced Opioid Policy Supported by Clinicians, Politicians, and the Media’ by William Scholten and Jack E. Henningfield [87]. The correct Declaration appears below. The publisher apologizes for this error”. This new Declaration excluded the claim that no COIs existed.

The healthcare community should demand the highest levels of transparency and disclosure in published studies before the claims of those studies are incorporated into clinical practice, especially those funded by industry. Vague statements such as a ghostwriter providing “editorial assistance” or academic authors retaining “editorial control” should not reduce scrutiny of potential COIs. The evaluation of COIs should be based on the full disclosure of all financial relationships and potential COIs, including detailed reporting of all relevant financial interests, activities, relationships, and affiliations [85]. Authors should fully disclose the extent to which medical writers contributed to published papers. Finally, given the lessons from the opioid epidemic, governments and academia should consider shifting from private sector partnerships to a norm of separation and develop counterstrategies that insulate themselves from corporate influence and preserve their integrity and public trust [88].

### 4.6. Limitations

There are several limitations to this study. The OIDA is a dynamic repository containing millions of industry documents that are continuously updated as new legal cases are settled. As such, our analysis was limited to the documents available at the time of the study. Nevertheless, given that J&J was a defendant in the Oklahoma litigation, which included an examination of IPPC activities, it is reasonable to believe that the relevant documents were accessible for our review. Additionally, because the IPPC was disbanded during associated litigation, we were only able to analyze some of their stated goals rather than the actual impact of these initiatives. Lastly, while we contextualized IPPC activities within the broader body of the literature on opioid marketing to enhance scientific rigor, the supporting literature cited is not intended to be exhaustive. However, the narrative review was designed to reflect the dominant themes and findings of the literature.

## 5. Conclusions

The findings of this study underscore the pharmaceutical industry’s role in contributing to the opioid crisis through targeted marketing strategies, specifically toward U.S. military veterans. Janssen Pharmaceuticals’ Imagine the Possibilities Pain Coalition (IPPC) exemplifies how industry-funded initiatives sought to increase opioid prescribing to veterans by downplaying addiction risks and overstating benefits. These tactics, which included ghostwriting scientific literature and leveraging empathetic messaging, reveal the potential for industry influence to undermine evidence-based care. For the healthcare community worldwide, this study highlights the importance of prioritizing unbiased practices, ensuring transparency in public health initiatives, and scrutinizing educational materials for potential conflicts of interest. Addressing these concerns is crucial to protecting vulnerable populations like veterans and preserving trust in healthcare institutions. These findings also call for robust counterstrategies to insulate clinical practice and medical education from corporate influence, thereby supporting more sustainable and ethical healthcare policies. The Supplementary Materials (see Supplementary File S1: Responsible Conduct of Research Case Studies) include three responsible conduct of research (RCR) case studies intended for use in journal clubs, ethics committee discussions, continuing medical education (CME) sessions, residency programs, professional development workshops, and multidisciplinary team meetings. These RCR case studies are based on information from this article and are a companion piece.

## Data Availability

The industry documents included in this study are available through the University of California, San Francisco Opioid Industry Document Archive (OIDA): https://www.industrydocuments.ucsf.edu/opioids (accessed on 10 December 2024). Instructions for identification and bulk download of the OIDA documents through Johns Hopkins University are outlined in the Methods Section.

## Supplementary Materials

The following supporting information can be downloaded at  https://www.mdpi.com/article/10.3390/healthcare13040434/s1, Supplementary File S1: Responsible Conduct of Research Case Studies.
