# Correction: Haddock et al. *Imagine the Possibilities Pain Coalition* and Opioid Marketing to Veterans: Lessons for Military and Veterans Healthcare. *Healthcare* 2025, *13*, 434

**DOI:** 10.3390/healthcare14121653

**Published:** 2026-06-11

**Authors:** Christopher K. Haddock, Luther Elliott, Andrew Kolodny, Christopher M. Kaipust, Walker S. C. Poston, Jennifer D. Oliva, Eleanor T. Lewis, Elizabeth M. Oliva, Nattinee Jitnarin, Chunki Fong

**Affiliations:** 1 NDRI-USA, Inc., 1920 West 143rd Street, Suite 120, Leawood, KS 66224, USA; kaipust@ndri-usa.org (C.M.K.); poston@ndri-usa.org (W.S.C.P.); jitnarin@ndri-usa.org (N.J.); fong@ndri-usa.org (C.F.); 2 School of Global Public Health, New York University, 708 Broadway, 6th Floor, New York, NY 1003, USA; luther@nyu.edu; 3 Heller School for Social Policy and Management, Brandeis University, 415 South Street, Waltham, MA 02453, USA; akolodny@brandeis.edu; 4 Maurer School of Law, Indiana University, 211 S. Indiana Ave., Bloomington, IN 47405, USA; jenoliva@iu.edu; 5 Program Evaluation and Resource Center, Center for Innovation and Implementation, Department of Veterans Affairs, 795 Willow Road, Menlo Park, CA 94025, USA; eleanor.lewis@va.gov (E.T.L.); elizabeth.oliva@va.gov (E.M.O.)

**Error in Conflicts of Interest Statement**

There was an error in the original publication [1]. The statement that the authors declare no conflicts of interest was published before a disclosure from Andrew Kolodny was received and is incorrect. Andrew Kolodny requested that the record be corrected. The corrected statement of conflicts of interest appears below:

**Conflicts of Interest:** Christopher K. Haddock, Christopher M. Kaipust, Walker S. C. Poston, Nattinee Jitnarin and Chunki Fong are employed by the National Development & Research Institutes, Inc. (NDRI-USA, Inc.). Andrew Kolodny has received fees for serving as an expert witness in litigation against the opioid industry and is the president of the nonprofit organization Physicians for Responsible Opioid Prescribing. The other authors have nothing to declare.

The authors state that the scientific conclusions are unaffected. This correction was approved by the Academic Editor. The original publication has also been updated.
