# Supplementary material for: Imagine the Possibilities Pain Coalition and Opioid Marketing to Veterans: Lessons for Military and Veterans Healthcare
Source: Healthcare (Basel). 2025 Feb 18;13(4):434. doi: 10.3390/healthcare13040434 (PMC11855145; doi:10.3390/healthcare13040434)
Supplement: Supplementary file 1 [file healthcare-13-00434-s001.zip › healthcare-3394855-supplementary.pdf]

Responsible Conduct of Research Case Study Guide: Ethical Challenges in Writing and Consuming Industry-Sponsored Research

Balancing Integrity and Influence: Ethical Considerations in Producing and Interpreting Industry Sponsored Studies from the Opioid Epidemic

---

Introduction: To fully engage with this case study, we encourage readers to examine the following two papers and reflect on the key lessons they provide for Responsible Conduct of Research (RCR):

Haddock, J. et al. (date). ***Targeting Veterans: The "Imagine the Possibilities Pain Coalition" and Opioid Marketing.***

The Haddock et al paper highlights the pharmaceutical company's marketing strategies that targeted vulnerable populations, including veterans, to promote opioid products. It demonstrates how corporate practices leveraged incomplete or misleading scientific evidence to downplay risks and overstate benefits, influencing clinical practice and public health outcomes. This paper underscores the ethical and methodological challenges in industry-funded studies, particularly concerning bias, selective reporting, ghostwriting, and the manipulation of data to support commercial objectives.

Gac, B. W., Yakubi, H., & Apollonio, D. E. (2023). ***Issues arising from the study design, conduct, and promotion of clinical trials funded by opioid manufacturers: a review of internal pharmaceutical industry documents.*** **Evidence & Policy, 19(4), 536-553. Retrieved Jan 20, 2025, from https://doi.org/10.1332/174426421X16856230946027.**

This article examines biases in the design, conduct, and reporting of clinical trials funded by opioid manufacturers. It highlights how enriched enrollment study designs, selective reporting, and professional conflicts of interest compromise the validity and transparency of research findings. These issues are highly relevant to RCR, as they illustrate threats to both internal and external validity and emphasize the need for critical appraisal of industry-sponsored studies.

Discussion:

1. **Transparency**: How do we ensure that all contributions, including industry-funded ghostwriting, are fully disclosed to surface potential bias and maintain credibility?
2. **Bias Mitigation**: What safeguards can researchers engaged in research within industry or partnered with industry implement to minimize selective reporting or manipulation of data?
3. **Accountability**: How can healthcare providers critically evaluate industry-sponsored studies to ensure unbiased evidence informs clinical decisions?

Scenario: A pharmaceutical company sponsors a review article on a new opioid treatment for chronic pain. The company's proposal includes providing editorial assistance through a medical communications firm. Simultaneously, healthcare providers must decide whether to integrate findings from industry-sponsored trials into practice guidelines.

Interactive Exercise:

1. Assign participants to roles: a pharmaceutical company representative, a researcher writing the review, and a clinician evaluating the research.
2. Debate the following:
   - Should ghostwriting support be accepted if it ensures timely publication? What can authors do to minimize the potential ethical issues with ghostwriting?
   - How can healthcare providers identify and address potential biases in the findings?
3. Select and review an abstract from a pharmaceutical-funded trial of an opioid drug, identifying areas of potential bias, incomplete disclosure, or positive examples of appropriate disclosure.

Learning Objectives:

- Understand ethical challenges in collaborating with industry.
- Develop strategies to ensure transparency and accountability in authorship.
- Enhance critical appraisal skills for interpreting sponsored research.

Real-World Takeaway: This case underscores the importance of fostering transparency and critical evaluation in the production and consumption of industry-sponsored studies to protect public health.

Responsible Conduct of Research Case Study Guide: Authoring Industry-Sponsored Research

Navigating Authorship and Integrity in Pharmaceutical Industry Research

---

Introduction: To fully engage with this case study, we encourage readers to examine the following two papers and reflect on the key lessons they provide for Responsible Conduct of Research (RCR):

Haddock, J. et al. (date). ***Targeting Veterans: The "Imagine the Possibilities Pain Coalition" and Opioid Marketing.***

The Haddock et al. paper highlights the pharmaceutical company's marketing strategies that targeted vulnerable populations, including veterans, to promote opioid products. It demonstrates how corporate practices leveraged incomplete or misleading scientific evidence to downplay risks and overstate benefits, influencing clinical practice and public health outcomes. The paper underscores the ethical and methodological challenges in industry-funded studies, particularly concerning bias, selective reporting, ghostwriting, and the manipulation of data to support commercial objectives.

Gac, B. W., Yakubi, H., & Apollonio, D. E. (2023). ***Issues arising from the study design, conduct, and promotion of clinical trials funded by opioid manufacturers: a review of internal pharmaceutical industry documents.*** **Evidence & Policy, 19(4), 536-553. Retrieved Jan 20, 2025, from https://doi.org/10.1332/174426421X16856230946027.**

This article examines biases in the design, conduct, and reporting of clinical trials funded by opioid manufacturers. It highlights how enriched enrollment study designs, selective reporting, and professional conflicts of interest compromise the validity and transparency of research findings. These issues are highly relevant to RCR, as they illustrate threats to both internal and external validity and emphasize the need for critical appraisal of industry-sponsored studies.

Ethical Issues:

1. **Authorship Integrity**: How can researchers ensure credit and responsibility are appropriately assigned?
2. **Conflict of Interest**: What disclosures are necessary to maintain trust in research? If employees of pharmaceutical companies serve as authors, what disclosures should be made in the paper?
3. **Research Rigor**: How can authors prevent selective reporting or data manipulation?

Scenario: A researcher is offered substantial financial support from a pharmaceutical company to co-author a review article but is required to use text drafted by a medical writing firm hired by the company. The researcher must decide how to balance the benefits of the funding with the risks to credibility.

Interactive Exercise:

1. Participants role-play the decision-making process:
    - A researcher evaluating whether to accept ghostwriting support.
    - An industry representative advocating for efficiency through medical writing firms.
    - A journal editor assessing the disclosure requirements for such collaborations.
2. Evaluate a draft manuscript for transparency and rigor, identifying areas where disclosures or revisions are necessary.

Learning Objectives:

- Recognize ethical challenges in accepting industry support.
- Develop best practices for disclosing conflicts of interest.
- Strengthen strategies for maintaining research integrity.

Real-World Takeaway: This case emphasizes the importance of transparency and accountability to maintain public trust in industry-supported research.

Responsible Conduct of Research Case Study Guide: Consuming Industry-Sponsored Research

Critical Evaluation of Industry-Sponsored Papers

Introduction: To fully engage with this case study, we encourage readers to examine the following two papers and reflect on the key lessons they provide for Responsible Conduct of Research (RCR):

Haddock, J. et al. (date). ***Targeting Veterans: The "Imagine the Possibilities Pain Coalition" and Opioid Marketing.***

The Haddock et al. paper highlights the pharmaceutical company's marketing strategies that targeted vulnerable populations, including veterans, to promote opioid products. It demonstrates how corporate practices leveraged incomplete or misleading scientific evidence to downplay risks and overstate benefits, influencing clinical practice and public health outcomes. The paper underscores the ethical and methodological challenges in industry-funded studies, particularly concerning bias, selective reporting, ghostwriting, and the manipulation of data to support commercial objectives.

Gac, B. W., Yakubi, H., & Apollonio, D. E. (2023). *Issues arising from the study design, conduct, and promotion of clinical trials funded by opioid manufacturers: a review of internal pharmaceutical industry documents.* Evidence & Policy, 19(4), 536-553. Retrieved Jan 20, 2025, from https://doi.org/10.1332/174426421X16856230946027.

This article examines biases in the design, conduct, and reporting of clinical trials funded by opioid manufacturers. It highlights how enriched enrollment study designs, selective reporting, and professional conflicts of interest compromise the validity and transparency of research findings. These issues are highly relevant to RCR, as they illustrate threats to both internal and external validity and emphasize the need for critical appraisal of industry-sponsored studies.

Ethical Issues to Discuss:

1. **Bias Detection**: How can healthcare providers identify subtle biases in sponsored studies?
2. **Evidence-Based Practice**: How should providers weigh industry-sponsored findings against independent research?
3. **Patient Advocacy**: How can providers ensure patient care is informed by unbiased evidence?

Scenario: A clinician is reviewing a pharmaceutical industry-funded research paper that evaluates the efficacy of a new opioid medication. This paper has been published in a reputable journal, but there are concerns about potential ethical and methodological issues that could compromise its validity.

Interactive Exercise:

1. **Analyze Hypothetical Research Excerpts:** Provide participants with fictional excerpts from the paper, such as the study's abstract, methods, and conclusions.
    - *Abstract:* "This randomized controlled trial demonstrates that the new opioid provides superior pain relief compared to alternatives, with minimal side effects."
    - *Methods:* "The study was designed by the sponsor, and all data analysis was performed by a third-party research organization contracted by the sponsor."
    - *Results:* "Relevant data supporting these conclusions are on file with the sponsor and available upon request."

    Ask participants to identify and discuss potential ethical issues they should consider, such as the following:

- Ghostwriting (e.g., lack of transparency about who authored the paper).
- Conflicts of interest (e.g., sponsor involvement in study design and analysis). o Missing or exaggerated data (e.g., selective reporting of results or lack of data availability).

2. **Evaluate Ethical and Methodological Concerns:**
   - What ethical issues arise when the sponsor designs the study or controls access to data?
   - How might ghostwriting affect trust in the research process and the accuracy of the conclusions?
   - Discuss how omitting relevant data or exaggerating findings can mislead clinical practice and patient safety.

3. **Apply Ethical Decision-Making:**
   - Brainstorm steps clinicians can take to critically evaluate the credibility of industry-funded research papers, such as the following:
     - Checking for independent replication of results.
     - Reviewing conflicts of interest disclosures.
     - Identifying whether the methodology adheres to best practices (e.g., blinding, transparency in data sharing).
   - Explore how clinicians can prioritize evidence-based care in the face of potential bias.

4. **Discuss Broader Implications:**
   - Facilitate a discussion on how unethical practices in research can erode public trust in medicine.
   - Ask participants to propose strategies for addressing these issues, such as promoting stricter journal policies on transparency and ensuring that clinicians are trained to critically appraise research.

Learning Objectives:

- Enhance skills in identifying bias and incomplete evidence.
- Understand the role of industry influence in clinical guidelines.
- Promote patient-centered decision-making.

Real-World Takeaway: This case highlights the importance of critically appraising industry-sponsored research to ensure unbiased, evidence-based healthcare.
